# The ecology of human fear: survival optimization and the nervous system

**DOI:** 10.3389/fnins.2015.00055

**Published:** 2015-03-18

**Authors:** Dean Mobbs, Cindy C. Hagan, Tim Dalgleish, Brian Silston, Charlotte Prévost

**Affiliations:** ^1^Department of Psychology, Columbia UniversityNew York, NY, USA; ^2^Medical Research Council-Cognition and Brain Sciences UnitCambridge, UK

**Keywords:** survival optimization system, defensive distance, fear, anxiety, periaqueductal gray, amygdala, appraisal

## Abstract

We propose a Survival Optimization System (SOS) to account for the strategies that humans and other animals use to defend against recurring and novel threats. The SOS attempts to merge ecological models that define a repertoire of contextually relevant threat induced survival behaviors with contemporary approaches to human affective science. We first propose that the goal of the nervous system is to reduce surprise and optimize actions by (i) predicting the sensory landscape by simulating possible encounters with threat and selecting the appropriate pre-encounter action and (ii) prevention strategies in which the organism manufactures safe environments. When a potential threat is encountered the (iii) threat orienting system is engaged to determine whether the organism ignores the stimulus or switches into a process of (iv) threat assessment, where the organism monitors the stimulus, weighs the threat value, predicts the actions of the threat, searches for safety, and guides behavioral actions crucial to directed escape. When under imminent attack, (v) defensive systems evoke fast reflexive indirect escape behaviors (i.e., fight or flight). This cascade of responses to threat of increasing magnitude are underwritten by an interconnected neural architecture that extends from cortical and hippocampal circuits, to attention, action and threat systems including the amygdala, striatum, and hard-wired defensive systems in the midbrain. The SOS also includes a modulatory feature consisting of cognitive appraisal systems that flexibly guide perception, risk and action. Moreover, personal and vicarious threat encounters fine-tune avoidance behaviors via model-based learning, with higher organisms bridging data to reduce face-to-face encounters with predators. Our model attempts to unify the divergent field of human affective science, proposing a highly integrated nervous system that has evolved to increase the organism's chances of survival.

## Introduction

Charles Darwin declared that organisms unable to adapt to the demands of their environment will fail to pass on their genes and consequently fall as casualties in the “war of nature” (Darwin, 1871). One of the most pervasive ecological demands is predatory avoidance. The relentless pressure to outwit predators while balancing homeostatic threats, such as resource depletion, has produced a nervous system that optimizes survival actions. These optimal actions provide the organism with a survival intelligence that permits appropriate responses to an array of environments and circumstances that range from non-threatening to life endangering. In humans this behavioral repertoire is supported by a neurobiological system that has endowed us with a powerful set of intelligent survival mechanisms, promoting adaptation to changing ecologies and efficient navigation of natural dangers. Building off of a set of well-known neural systems, recent insights from human and comparative neuroscience have demonstrated that midbrain regions are involved in phylogenetically older adaptations such as reflexive fight, flight, or freeze (FFF) systems and are strongly interconnected, innervated by, and in competition with, forebrain structures including the prefrontal cortex (PFC) and amygdala (Öngür et al., 2003; Price, 2005; Mobbs et al., 2007; Price and Drevets, 2010).

Innovations in the field of affective neuroscience offer new insights on existing theories concerning the phylogenetic continuity and discontinuity of survival responses from lower animals to humans (Panksepp, 1998, 2005; McNaughton and Corr, 2004; Woody and Boyer, 2011; Adolphs, 2013). Importantly, while many of these models have focused on rodent responses to threat, our human ancestors were under many of the same selection pressures common to all other animals, yet the cross-applicability of these hard-wired systems to human threat response, including fight, flight, freezing and startle reactions has often been overlooked. However, unique pressures may also play a role in human survival intelligence—these extend from cunningness, an understanding of others' sinister desires, and metacognitive processes including meta-strategic knowledge (Zohar and David, 2009). Thus, higher-level and more integrated cognitive and computational systems embedded in our nervous system are likely to be critical to human survival. A compelling narrative on the survival systems that are evoked to protect the human organism against predatory threat must therefore be multi-disciplinary, encompassing ideas not only from the provinces of behavioral ecology, ethology and evolutionary biology but also from behavioral, social, cognitive and computational neuroscience.

In this article, we gather ecological theories and empirical data from a variety of related fields in an attempt to create a unified model of how humans predict, respond to, control and learn about danger. We will discuss hierarchical theories of threat, how these hierarchies are represented within a global neural architecture, and present evidence for components of an optimized survival system in humans, which is consistent with (c.f. Blanchard et al., 2011), and sometimes distinct from those observed in other animals. The Survival Optimization System (SOS) is based on the assumption that a set of systems have evolved to avoid and combat threats that pose danger to the species' fitness. These extend from neurocognitive systems that predict the sensory environment, orienting toward potential threat, assessing threat and escape possibilities, and to hard-wired defensive reactions instantiated by the oldest sectors of the nervous system. In tandem, these survival strategies are steered by modulatory systems including cognitive appraisal and learningsystems.

## Survival in nature

Natural selection increases fitness by optimizing survival relevant behaviors within a given species' environment. We therefore define survival intelligence as the organism's ability to master its environment by minimizing local threats and adapting to novel threats in changing ecologies. Responses to predatory threat depend on both flexible and fixed behavioral traits. The presence of fixed traits implies that predatory threat is pervasive and expressed across a species' evolutionary time (Nonacs and Dill, 1993), hard-wired and developed by gradualism or other evolutionary methods particular to the species (Gould and Eldredge, 1977). Flexible (or plastic) traits are associated with the ability to modify actions in the animal's ecological time (i.e., the animal's lifetime). As predicted by the theory of the “predatory-prey arms race,” these traits co-evolve in both predators and prey. Thus, an animal is continuously adapting to sustain its viability in an ever-changing ecological system (Van Valen, 1973). In perpetual fashion, changes in evolutionary time result in changes in ecological time and possibly vice versa via Baldwinian mechanisms (e.g., those species with greater capacity to learn new skills that enhance the ability to avoid predators will pass on their genes and avoidance skills). Although some theorists dispute such co-evolution in predators and prey (Rosenzweig et al., 1987), other evolutionary pressures (e.g., environmental stresses including climate change, disease, resource availability, and migrating alien species) have sculpted cognitive, behavioral and neurobiological phenotypes that form fast-track systems in the prey's perception, attention, and decision-making.

### Ecological models of predator-prey encounters

While behavior and outcomes in predator-prey interactions are likely to be more complex and prone to species-specific differences, Lima and Dill (1990) have proposed that the predation risk or probability of being killed over a certain time interval can be captured by the following equation:

P(getting killed)=1−exp (−αd T).

where α is the rate of predator and prey encounters, *d* is the probability of death given an encounter and T is the time spent in situations of predation risk. The authors propose that α, *d*, and T are the basic building blocks to predation risk and accessible to be used by the prey for its benefit. If the predator is predictable or stays in the same patch for long periods of time α will be low. Access to α may also be adjusted by territory markings, margins of safety or vicarious learning (Lima and Dill, 1990). Therefore, according to this model, minimizing the number of threat encounters, and reducing the level of danger and duration of these encounters will maximize survival.

**Figure 1:**
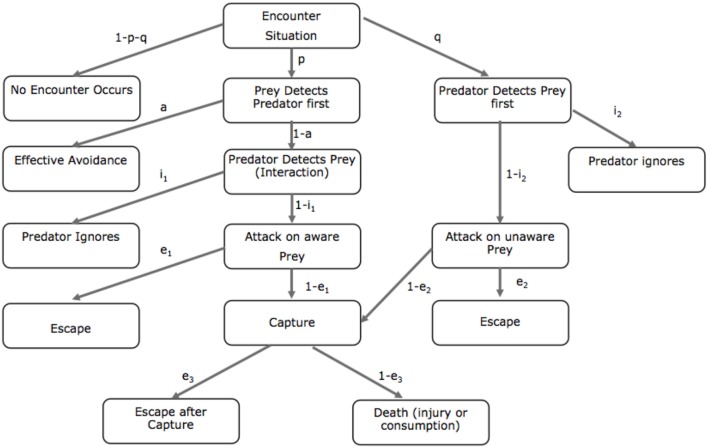
**Lima and Dill's predator-prey model**. Flow chart displaying possible outcomes of a predator-prey encounter. The symbols signify the conditional probabilities of each step of the pathway. *a*, avoid; *e*, escape; *I*, ignore; *p*, probability that the prey detects the predator first; *q*, probability that the predator detects the prey first (adapted from Lima and Dill, 1990).

While the behavioral options that control predation risk may still be unclear, Lima and Dill (1990) created a flow chart characterizing predator-prey encounters (Figure 1). The Lima and Dill model charts the interactive permutations that characterize predator and prey interactions and the probabilities of escape or death. The authors propose that the probability of death (*d*) associated with a predatory encounter can be denoted as a set of probabilities that an encounter with a predator will occur. Therefore, *d* can be defined as:

$$d=[p(1-a)(1-{\text{i}}_{1})(1-e_{1})+q(1-{\text{i}}_{2})(1-e_{2})](1-e_{3}).$$

The goal of the organism is to facilitate behaviors that enhance p (detect the predator first) and reduce detection by the predator (q). When the threat is distal, the animal tends to enhance *a* (e.g., freezing), whereas when the threat is attacking, and presumably proximal, the animal will facilitate flight/fight responses. Finally, if the animal is caught, fight and flight responses are supported by analgesic responses that suppress otherwise disabling pain and enhance escape behaviors. Lima and Dill (1990) suggest that the subcomponents of their equation (e_1_, e_2_) are known to the prey, including knowledge of escape routes, distance to predator and margin of safety. It is access to these subcomponents that allows the prey to have some behavioral control over risk. Furthermore, optimal prey survival involves the ability to spot the predator (*p*) before being spotted (*q*). Conversely, optimal predatory behavior involves attacking the unsuspecting prey (1 − *i*_2_). The goal of higher animals is to avoid such encounter situations by simulating the conditions to produce possible outcomes and reducing the likelihood of predator encounters.

### Species-specific and ecological factors

The survival strategies of the species determines how the probabilities are expressed in Lima and Dill's model. Niche-independent animals that navigate multiple habitats have evolved to successfully adapt to changing and unpredictable predators through the use of a plastic set of cognitive and behavioral systems. According to the “Predator Recognition Continuum Hypothesis,” prey that are exposed to a large variety of predators should display the greatest plasticity in responses to threat (Ferrari et al., 2007) and receive the greatest benefit from recognizing and identifying different types of predators. The particular calculations made by the prey are determined by the type of predator encountered. For human-human encounters, the calculations are likely to be complex. Our Pleistocene ancestors were masters at adapting to changing environments and solving problems under novel circumstances. Supporting this theory, the human brain is specialized in improvisation, where cells become tuned to adaptively code current information that is relevant to the present environment (Duncan, 2001). These cells essentially become multi-specialized, able to adapt to participate in different functions. This theory of adaptive coding is supported by research using non-human primate electrophysiology (Freedman et al., 2001) and suggests that the brain remains plastic in order to flexibly and optimally cope with a changing world. These cells are likely to be more numerous in humans and are probably located in the prefrontal cortex, as frontal regions are highly interconnected suggesting significant interaction and exchange, and are critical to the monitoring, correction, and control of behavior (Miller and Cohen, 2001).

Like other animals, humans make use of a reflexive set of systems to combat encountered threats that are neither predicted nor detected in time to be avoided. Fixed traits are innate, evolutionarily stable strategies that are widely prevalent across mammals including passive (e.g., freezing) and active defensives (i.e., fight or flight) that are evoked when the animal detects or engages a threat. Freezing is a simple yet powerful avoidance strategy that reduces motion and visibility, thereby facilitating and enhancing the gathering of information and increasing the possibility of disengaging the distal predator's attention. The proposed goal of flight is to increase the distance between the predator and prey, thereby increasing the chances of escaping. Finally, the fight response is a last resort when other strategies have been exhausted or no other strategy is feasible, such as when escape is not possible, and aims to discourage the predator from further attack (Blanchard and Blanchard, 1990a,b). These survival abilities may have developed in early niche-dependent animals, where a clear set of successful ecological rules governed simple fixed defensive responses.

While most mammals possess a subset of these flexible and fixed traits, the most salient feature of our framework is the speculation that modern day humans are particularly reliant on flexible systems that make use of sophisticated prediction (e.g., mental simulation) and learning strategies to avoid danger. Humans have a great capacity to learn what is important in changing environments and optimize behaviors accordingly to fulfill new goals (e.g., the importance of secondary reinforcers such as money). We speculate that the fidelity of both flexible and fixed neural survival circuits may equate to the extinction-resistance of the organism, and that flexible systems in humans permit our survival in the least optimal of circumstances. While differences between current and evolutionary environments suggest that fixed systems may be the source of problems (e.g., stress), we further speculate that a side effect of extensive flexibility (e.g., the ability to cognize about future oriented threat) may render humans vulnerable to psychopathology.

## Hierarchical models of survival

Darwin proposed that fear manifests along a gradient from attention to extreme terror (Darwin, 1871). Likewise, Kavaliers and Choleris (2001) suggest the existence of an “apprehension gradient” which extends from no interest to complete preoccupation with the predator. Some theorists differentiate fear from anxiety by proposing that fear results from the presence of highly imminent or tangible threat, while anxiety occurs when an aversive stimulus is abstract or remote in time or space (Rachman, 1979; Bouton et al., 2001). Likewise, research conducted by Blanchard et al. in rodents suggests three levels of danger; potential threat, distal threat, and proximal threat (Blanchard et al., 1986). Clinical researchers further differentiate fear into three separate components—the subjective apprehension of threat, physiological arousal, and behavior as attempts to avoid the threatening stimulus (Rachman, 1979). Contemporary theorists such as LeDoux (2012) propose that the concept “fear” is incorrectly used to capture a constellation of conscious feelings and behavioral and physiological responses, and this confuses and impairs progress in understanding the neural systems that underlie emotion. LeDoux's remedy is to examine “threat-induced defensive reactions” to distinguish feelings from brain/bodily responses, proposing that this bifurcation better reflects the way the brain has evolved and facilitates the assessment of similar processes in animal brains (Mobbs et al., 2009; Perkins et al., 2009; LeDoux, 2013).

LeDoux's sentiments are reflected in Fanselow and Lester's “Threat Imminence Continuum” model in which distinct threat-states change depending on whether a threat is absent, detected, or attacking (Fanselow and Lester, 1988). This continuum encompasses four core stages: (i) the Preferred phase is the time period when the animal is in a safe place, such as a nest or home dwelling. It is also assumed that the animal is free from homeostatic threats such as resource-depletion; (ii) The Pre-Encounter phase is the time period where the risk of threat is present, although there is no detectable presence of danger. An example would be a bird's move from the nest to the forest floor. The Pre-Encounter phase is therefore most evident when the animal is foraging for food or engaging in coitus. (iii) Post-Encounter threat is when a threat is detected, but there is no direct interaction between the prey and predator (e.g., the predator has not yet detected the prey) and (iv) Circa-Strike threat is the stage where the predator not only sees, but starts to pursue the prey with the intention of capture and consumption.

These different phases of imminence evoke stereotyped defensive behaviors in rodents, where the animal will choose strategies to prevent or defer its progression down the imminence continuum (Fanselow and Lester, 1988). Relative to the Preferred context, the Pre-Encounter phase is characterized by increased vigilance and arousal. Contextual fear conditioning may occur during subsequent encounters with the predator in the same context. Of course, species will differ in their defensive reactions (Bolles and Grossen, 1970). During the Post-Encounter phase, freezing behaviors and adaptive autonomic responses, such as increased sweating and piloerection are typically observed. One potential purpose of these responses may be to decrease body temperature, thereby thwarting (reptilian) predators that use heat sensitivity to detect prey. Most animals will, however, flee from an approaching or looming threat (Blumstein, 2006), particularly when a safe refuge is available (Blanchard and Blanchard, 1990a). On the other hand, humans may be more prone to exhibit fear responses that have evolved through our complex social interactions, as opposed to the stereotyped responses commonly observed in rodents.

If Post-Encounter behaviors fail, the animal will be pursued by the predator, evoking a switch to Circa-Strike defensive behaviors that are characterized by active coping strategies such as flight and near-contact fighting. The spatial distance to the threat may moderate these responses. For example, when a rat is placed in a box at a far distance from a cat, the rat will freeze or flee if there is an escape route, yet when the box is placed nearer to the cat, the rat will panic, displaying active flight or fight (Blanchard and Blanchard, 1990a) responses. Therefore, the threat context and distance to the threat are both crucial for eliciting particular defense strategies. The type of predator (e.g., bird or snake) also influences the prey's response. For example, the direction or velocity of the predator's approach (Fanselow and Lester, 1988) elicits different behavioral response strategies (e.g., crouching or freezing) by the prey. Furthermore, the predator's size and attack strategy plays a critical role in determining whether the prey is likely to engage in fight vs. flight behaviors.

Building off rodent and human clinical neuroscience, McNaughton and Corr (2004) proposed that two parallel defensive systems exist: (i) A defensive approach system, which is associated with approach anxiety during foraging (i.e., checking if a stimulus is a threat or food); (ii) A defensive avoidance system allied with panic or fear. The defensive avoidance system is typically engaged when a potential threat is moving closer or attacking. Blanchard and Blanchard have proposed that small distances result in explosive attack, whereas intermediate and large distances result in freezing and non-defensive behaviors, respectively. Another important element that determines which defensive strategy an organism selects is the perceived controllability of the situation (Maier and Watkins, 2005). Highly controllable situations are likely to elicit flight responses whereas uncontrollable or inescapable situations are likely to elicit immobility (Maier and Watkins, 2005). Survival intelligence therefore involves an adaptive fear system in which specific parts of the hierarchy are recruited as a function of the perceived intensity (e.g., proximity) of the threat.

## Neurohierarchical models of survival circuits

**Figure 2:**
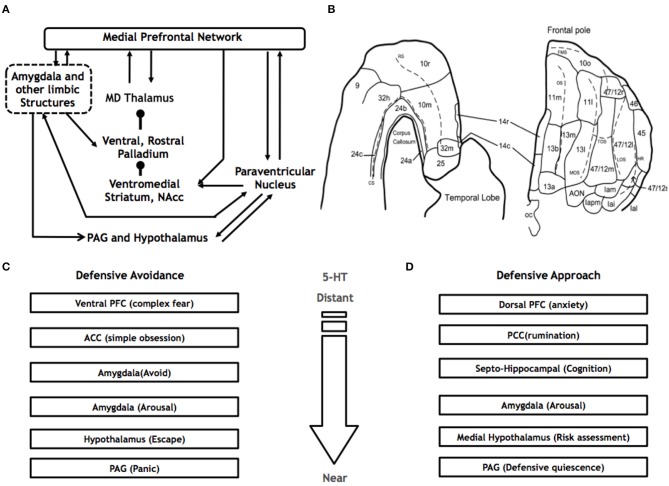
**The Medial PFC network**. **(A)** Based on non-human primate research, the major connections of the medial PFC, include the amygdala, PAG, hypothalamus, ventromedial striatum, pallidum and mediodorsal (MD) thalamus. **(B)** The medial PFC occupies the medial surface of the PFC, encompassing Brodmann's areas 32, 2, 14, 10, and 24 (adapted from Carmichael and Price, 1994). McNaughton and Corr's (2004) parallel neurocircuits of **(C)** defensive avoidance and **(D)** defensive approach.

Anatomical work suggests that the neurobiological systems that underlie the threat response are mapped along a hierarchical continuum (Figure 2). Based on non-human primate anatomy, Price has proposed that two core prefrontal cortical networks exist: (i) the medial prefrontal (mPFC) and (ii) the orbital prefrontal (OFC). The OFC network is conceptualized as a set of regions involved in multisensory processing such as taste, sight and sound (Price, 2005; Price and Drevets, 2010) and as such, is likely to be involved in the perception of threat. The interconnected mPFC network, however, is involved in the active experience of emotion. The mPFC network encompasses projections between, among other regions, the medial surface of the PFC, the amygdala, hypothalamus and periaqueductal gray (PAG). The presence of reciprocal connections between segments of the medio-dorsal thalamus and PFC, as well as robust afferent connections from the amygdala, supports this network's role in affective processing. Other thalamic nuclei, including the periventricular nucleus, receive projections from the dorsal PAG, and dorsal raphe nuclei (DRN) and are reciprocally connected to the amygdala and ventral striatum, supporting the role of the periventricular nucleus in stress responses (Price and Drevets, 2010). The mPFC network thus acts to regulate the experience of emotion, interacting with regions known to be involved in social perception, biological motion, social behavior, and memory including the superior temporal sulcus (STS), posterior cingulate cortex (PCC), and hippocampus. Recent work suggests a role for a salience network involving attentional focus mediated by norepinephrine and acetylcholine, and corticosteroids. Tonic norepinephrine activity originating from the locus coeruleus (LC) supports enhanced environmental scanning and reorientation of attention at the cost of selective task focused attention, and eliminates the attentional blink phenomena (Hermans et al., 2014). Attentional blink is the inability to detect the second of two quickly presented stimuli in succession and has been associated with phasic LC firing that leads to a refractory period in which subsequent LC responses are inhibited, and subjects are unable to detect the second stimuli. Above some affective threshold, activity in salience circuits that include LC, amygdala, and thalamus and their projections to PFC areas may impair PFC function in favor of midbrain, fixed systems resulting in rigid response type behavior. After this response is initiated, a slower acting corticosteroid response occurs, which eventually dominates and renormalizes the system. Canonical associative learning is enhanced during the acute stress period when norepinephrine activity peaks, while subsequent corticosteroid action serves to mute emotional effects and interference, reduce anxiety, and sympathetic arousal enabling the organism to bring flexible systems back online to optimize subsequent actions.

McNaughton and Corr (2004) have also proposed a set of neural circuits associated with defensive approach and defensive avoidance (Figures 2C,D). The defensive approach system extends from the dorsal prefrontal cortex → posterior cingulate → hippocampus → amygdala → medial hypothalamus → PAG. On the other hand, the defensive avoidance system is mediated by the ventral prefrontal cortex → anterior cingulate → amygdala → hypothalamus → PAG pathway. These neuroanatomical pathways are further supported by neuroanatomical research on non-human primates (Öngür et al., 2003; Price, 2005) and rodents (Fanselow, 1991, 1994; Bandler et al., 2000) and are also closely aligned with what Panksepp calls the FEAR circuitry (Panksepp, 2011)—the prosencephalic system propounded by Canteras et al. (2001). These fear circuits have been further clarified by the mapping of parallel circuits via the hippocampus, septum, hypothalamus and PAG (Gross and Canteras, 2012). Furthermore, distinct types of fear may have discrete pathways from the amygdala-hypothalamus and PAG (Gross and Canteras, 2012). LeDoux has recently proposed that such circuits assist in the survival of the organism by organizing brain functions that are optimized for adapting to different ecological threats (LeDoux, 2012).

In humans, the existence of these networks has been supported by brain imaging research using functional magnetic resonance imaging (fMRI) and employing an active avoidance paradigm where the goal was to actively evade an artificial predator with the capacity to chase, capture and shock the subject. Results showed that when the artificial predator is distant, increased activity is observed in the ventromedial prefrontal cortex (vmPFC). However, as the artificial predator moves closer, a switch to enhanced activation in the midbrain PAG is observed (Mobbs et al., 2009) This finding was later replicated and extended by showing that panic-related motor errors (wrong button presses resulting in collisions with the virtual walls of the maze) correlated with increased activity in the midbrain PAG and dorsal raphe nucleus (DRN); (Mobbs et al., 2009) as did placing a Tarantula progressively closer to the subject's foot while they were supine in an MRI scanner (Mobbs et al., 2010). The studies are supported by human diffusion tensor imaging showing white-matter connections between the midbrain and PFC (Hadjipavlou et al., 2006).

## Overview of the human survival optimization system

**Figure 3:**
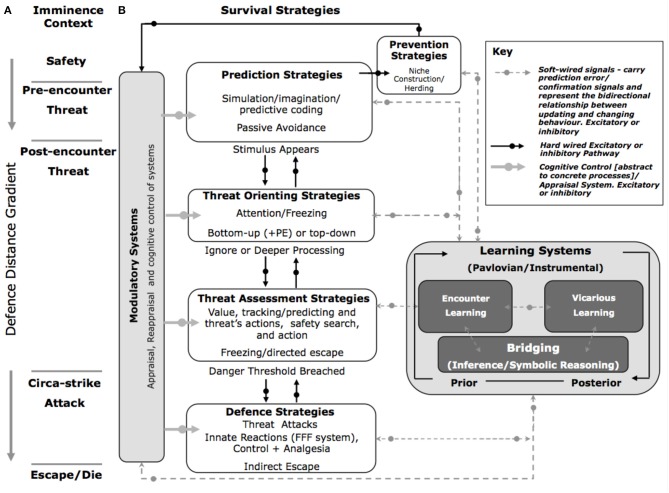
**The Survival Optimization System (SOS)**. **(A)** Fanselow and Lester's “Threat Imminence Continuum” **(B)** The SOS proposes that five strategy systems that are associated with predatory imminence and processed in a serial or non-serial manner. Each stage is modulated by several interacting systems including a cognitive system that selectively controls and regulates survival strategies along an abstract to concrete threat continuum. Internal probabilistic models subconsciously learn about, and facilitate responses to, threat. As one moves from predictive to defensive systems, innate and fixed systems are increasingly engaged. PE, prediction error; FFF, Flight/Fight and Freeze.

The long-term survival of the species depends on the ability to learn from, and optimally respond to, a potential or real threatening stimulus. We therefore propose five strategies that are central to survival: prediction; prevention; threat orienting; threat assessment; and rapid reaction to imminent danger at varying defensive distances (Figure 3). Furthermore, we propose that these strategies are influenced by (i) modulatory systems that directly up or down regulate the five survival strategies by actively reconfiguring the threat circuits. These include: cognitive appraisal/regulation, interoceptive contexts, and metabolic drives. Feeding into these strategies and modulatory systems are a set of (ii) learning systems that include the utilization of internal probabilistic models, and vicarious learning. These features distinguish the SOS from previous models (c.f. McNaughton and Corr, 2004; Blanchard et al., 2011; Woody and Boyer, 2011), for example, the security motivational model put forward by Woody and Boyer (2011). These authors suggest that the security motivational model is switched on when a stimulus is judged a potential danger. This results in elevated anxiety where the organism appraises the situation, and results in a set of security behaviors (motor and visceral). These behaviors are species specific. These features of the security motivational model clearly overlap with our model. However, there are several features that distinguish our model from others:

- No human behavioral model based on threat context, such as those laid out by Fanselow and Lester (1988) and Lima and Dill (1990) has been put forward.
- We further link these ecological models to the neuroanatomical circuits that underlie survival.
- We tie these neural circuits into three core domains of behavior: (i) “pre-encounter” avoidance (ii) “post-encounter” directed escape and (iii) circa-strike indirect escape.
- We also incorporate recent advances in cognitive neuroscience including recent work on cognitive reappraisal and cognitive control and how these can tune both learning and survival strategies.
- The SOS incorporates recent advances in learning theory (e.g., social learning, computational accounts of learning).

## Five survival strategies

### Prediction strategies

The conscious prediction and simulation of future threat occurs during preferred safety context and pre-encounter threat and allows animals to prepare for, and flexibly attend to, potential danger. In humans, the highly developed ability to envisage, simulate and predict future scenarios allows us to modify our current behavior to prepare, escape, or even avoid possible future dangers (Suddendorf and Corballis, 2007). The prediction of threat results in precautionary behaviors, such as increasing alertness, environmental surveillance and what we call pre-encounter avoidance (i.e., avoiding threat before it is encountered). Furthermore, the prediction system results in prevention strategies, such as niche construction (Odling-Smee, 2003), risk dilution through living in the safety of groups, building safe havens (e.g., defensive walls or fortress) or constructing weapons. Therefore, a core goal of the prediction system is to avoid the encounter situation via prevention planning or avoidance (Figure 1).

While basic predictions occur through simple Pavlovian stimulus-response learned associations and instrumental actions, complex predictions involving imagination, simulation, and analogical reasoning, may, in terms of extent, be most enhanced in humans. Prediction can occur through very basic environmental “what if” cues (Moulton and Kosslyn, 2009) when escape routes are minimal or opaque (how difficult or easy it may be to escape a potential threat). Prediction formulations allow humans to use memories of past events to simulate or imagine the future and to consider what steps one might take in order to optimize escape from a threat (Hassabis et al., 2007). Imagination potentially allows one to remotely simulate unpredictable predatory behavior while minimizing predatory contact and energy exertion, thereby increasing the likelihood of escape.

- *Simulation and imagination*. Although rodents seem to show rudimentary forms of simulation (e.g., replay), the ability to simulate and envisage the future may be uniquely human in its complexity and critical to human survival (Corballis, 2013). One benefit of prospective systems is the ability to anticipate threat as well as highlighting escape strategies without experiencing actual danger. Simulation allows us to foresee and pre-live danger that is presently absent (Suddendorf and Corballis, 2007). Episodic memory stores personal memories about everyday experiences and therefore is critical to simulation and higher forms of prediction (Hassabis and Maguire, 2009). Episodic memory is not only crucial for remembering past events, but also supports so-called mental time travel, whereby humans both counterfactually remodel the past, and foresee, plan and change the future (Suddendorf and Corballis, 2007). While not a universal ability, mental time travel has been demonstrated in Great Apes (Mulcahy and Call, 2006) and Corvids (Dally et al., 2006), and is known to be particularly sophisticated in humans. Recent studies have shown that thinking about future consequences of one's decisions can influence current decision-making (Peters and Buchel, 2010) and it is possible that future-oriented survival encoding produces superior memory retrieval for survival-relevant information (Nairne et al., 2007; Klein et al., 2010).
- *Predictive coding*. Predictive coding is the theoretical proposal that the brain actively infers sensory input. Consequently, humans can efficiently disambiguate present from future information, thus allowing faster and optimal responses to threat. Summerfield et al. (2006) used fMRI to show increased top-down connectivity from the PFC to the fusiform gyrus during anticipation of forthcoming facial stimuli. These findings were interpreted as evidence that the medial PFC resolves perceptual ambiguity by matching what is expected or predicted to what is observed. Later research replicated these findings, further demonstrating that predictive coding is associated with activity within the medial PFC (Summerfield and Koechlin, 2008). These findings suggest that the brain makes active inferences about the sensory experiences we are likely to encounter. The formulation of predictions by the brain may serve to make quicker and more accurate decisions about potential danger in the environment.

### Prevention strategies

During the preferred safety state and pre-encounter threat, the ability of an organism to make accurate predictions concerning potential threat is highly adaptive and enables it to flexibly change behaviors to protect itself from future predation. We propose that prevention takes two forms:

- *Niche construction*. Humans, and many other animals, deploy various prevention strategies to reduce threats, including alteration of the environment. Put simply, if it is possible to predict a predatory attack, it is often possible to prepare for it in advance. This process, referred to as positive niche construction, (Odling-Smee, 2003) involves active alteration of the environment to increase the likelihood of survival. Animals will alter their environment by building nests, burrows or tree holes to protect themselves and their offspring. Rodents also show an increased fear of open areas (e.g., elevated plus maze, Pellow et al., 1985) and prefer to hide under ground. Humans utilize advanced technology and cooperation to optimize prevention strategies both in terms of scale and effectiveness by building large walls and fortresses, living inside safe homes and constructing prisons to contain the threat from dangerous criminals. Humans are more flexible and ingenious in the implementation of prevention strategies and may do so without any reason or in response to threats that may be encountered in the future. The knowledge that one is safe reduces the stress of predation, allows for delayed dispersion of the offspring and allows for activities that might otherwise be risky to survival.
- *Group living*. Evolutionary theorists have proposed that one key reason why animals live in groups is to protect themselves from predation. For example, Hamilton's Selfish-Herd hypothesis predicts that aggregations emerge from the organism's attempt to avoid predators resulting in a type of social gravity whereby individuals move toward other members (Hamilton, 1971). This theoretical stance has been supported by studies showing risk dilution (i.e., reduced probability of attack when in groups; Foster and Treherne, 1981), increased vigilance to threat (i.e., the many eyes hypothesis; Roberts, 1996) and group aggression where individuals cooperatively attack or harass a predator (Kruuk, 1964; Dominey, 1983). Therefore, group living is a key prevention strategy resulting in increased protection from threat.

### Threat orienting strategies

Detection of a potential threat initiates the post-encounter phase, which in turn instigates a set of predictable behavioral strategies. These strategies involve biological systems that have evolved to orient toward salient events (Ohman et al., 2001; LeDoux, 2012), and typically coincide with freezing. Mogg and Bradley (1998) propose that the goal of attention is to facilitate the detection of danger, and these threat biased attentions may take several forms. Todd et al. have characterized this further by proposing what they call an “affective control setting” which is a habitual mental set that favors motivationally relevant stimuli in a given context and that “affective biased attention” allows us to attend to certain categories over others. These authors further propose that this may be a form of emotion regulation. Affective-biased attention is driven by bottom-up systems, prone to learning and acts as a filtering process tuned and retuned over development (Todd et al., 2012). Attention therefore, may, be driven both by a fast, hard-wired system as well as by a sophisticated top-down filtering mechanism supported by predictions.

#### Attention and vigilance

Threatening stimuli cause on-going behaviors to cease, and lead to freezing and the orientation of attention toward the threat (Blanchard et al., 2011). Heightened vigilance is costly in terms of energy, loss of foraging opportunities, and disruption to ongoing goal-directed behavior (Eysenck, 1992). Vigilance is therefore fleeting and restricted to times when high-risk situations are predicted (Lima and Bednekoff, 1999). An advantage of the prediction system is that it allows the animal to efficiently direct the vigilance system to detect danger early, disambiguate stimuli, and ignore less salient information. Heightened vigilance is accompanied by tonic catecholaminergic action that increases environmental scanning and sensory signal processing at the expense of selective filtering (Sara and Bouret, 2012). While the signals processed contain more noise, broader sampling likely increases the probability of detecting potentially relevant stimuli outside of the current task set and represents a conservative strategy to detect threat. The prediction system may act as a balancing mechanism to optimize behavioral selection and reduce the disadvantage of noisy signals. Early detection via increased vigilance provides for additional time allowing consideration of different strategies to escape threat. Moreover, different pathways within the visual system may be engaged by different types of threat, with magnocellular pathways suggested to process low spatial frequency visual information and parvocellular pathways suggested to process high spatial frequency visual information. Interestingly, research has shown that the pulvinar and superior colliculus become active to fearful faces presented at low spatial frequency but not high spatial frequency, suggesting their exclusive involvement in the magnocellular pathway and in accordance with their potential to provide “quick and dirty” information to the amygdala about threat in the surrounding environment (Vuilleumier et al., 2003).

#### Biologically prepared to detect biological stimuli

Brain imaging studies of attention to threat have been employed to examine the neural substrates of emotion processing in humans, and typically implicate the amygdala. Pictures of angry faces evoke increased activity in the amygdala and parietal cortex which suggests their involvement in attention to danger signals (Mohanty et al., 2009). Ventrolateral PFC and the temporo-parietal junction (TPJ), on the other hand, are proposed to be involved in directing attention toward novel or new stimuli (Sylvester et al., 2013). High levels of anxiety in humans may predispose one to possess a greater attentional bias to threatening or potentially threatening stimuli. In support of the role of the amygdala as an emotional saliency detector, damage to the amygdala has been shown to attenuate the processing of rapidly presented aversive words shown in quick succession, known as the attentional blink. Measures of state anxiety have been shown to positively correlate with frontal and amygdala activity during the presentation of threatening faces (Bishop et al., 2004b). Increased amygdala activation to fearful faces has also been observed in individuals scoring high on measures of trait anxiety (Ewbank et al., 2009), patients with generalized anxiety disorder (Etkin et al., 2004; Etkin and Wager, 2007) and soldiers suffering from combat stress (van Wingen et al., 2011). Interestingly, highly anxious subjects (Bishop et al., 2004a) and patients with PTSD (Shin et al., 2005) also show marked disruption in the prefrontal cortex. In conjunction with the prefrontal cortex, the amygdala may work to cue an individual to environmental signals of interest (Schafer and Moore, 2011).

#### Reducing surprise

In the case of unpredicted danger, a prediction error occurs, triggering the engagement of the attention system and providing the animal with rudimentary information about the threat. The individual is given little if any time to prepare the most optimal course of action. Cortical regions such as the inferior parietal cortex are known to be involved in attentional processing directed toward salient and novel environmental stimuli (Gottlieb and Balan, 2010). The directed attention system is also influenced by midbrain regions, including the superior colliculus (Knudsen, 2011), parietal cortex, pulvinar and motor responding areas (Aston-Jones and Cohen, 2005). The superior colliculus is a crossmodal structure with motor layers that prioritize locations based on stimulus saliency and motor goals (Fecteau and Munoz, 2006), and cholinergic and GABAergic circuits involved in spatially selective enhancement of attention (Knudsen, 2011). The superior colliculus relays information to the forebrain about salient events (Knudsen, 2011). Midbrain attentional capture systems are therefore mechanisms that help to protect against the predator's stealth and surprise. Threat orienting is clearly broader than just attention and involves the whole cognitive system including perception, attention, and memory. Relatively unpredicted threats are likely to cause higher levels of anxiety/fear to manifest bringing about a stronger system-wide cognitive reconfiguration to orient to the threat. Individual differences at this level would mirror the processes at the Prediction System level with, for example, anxious people having a higher incidence of false alarms or bias because the system is configured conservatively (Mogg and Bradley, 1998).

### Threat assessment strategies

During threat assessment one needs to evaluate the context in which the threat is encountered and generate an appraisal of danger (Blanchard et al., 2011). Human crisis management theory proposes that responses to extreme threat include assessing a negative event, determining a response option and evaluating the utility of the response option (Sweeny, 2008). We propose that there are several sub-stages (some functioning in parallel) involved in assessing and acting upon information during post-encounter threat:

- *Post-encounter freezing*. When a potential threat is encountered, the first behavioral response of the animal will be to freeze—a form of passive coping, defined as an absence of all behavior except respiration (Bolles and Collier, 1976). Freezing allows for improved risk assessment by the animal and reduced detection by the predator, which in turn allows more time to make the optimal response. Freezing behaviors are instigated by the ventral columns of the PAG (vPAG) (Bandler et al., 2000). Defensive freezing occurs when a threat is detected and, in instances such as these, the amygdala and extended systems may compile relevant environmental information and signal the degree of threat to the vPAG (Fanselow, 1991). Indeed, efferents from the medial central nucleus of the amygdala (CeA) to the vlPAG are not only critical to instigating freezing, but also to suppressing other motivated behaviors such as foraging and mating (LeDoux, 2000).
- *Threat monitoring*. Threat monitoring is likely to engage yet another set of neural systems that includes, but is not limited to, the PFC and amygdala. One additional proposed region is the bed nucleus of the stria terminalis (BNST). The BNST is an important regulator of the hypothalamic-pituitary-adrenal (HPA) stress axis (Georges and Aston-Jones, 2002) and lesions of the BNST decrease anxiety (Duvarci et al., 2009). The BNST is implicated in threat monitoring and vigilance and strongly interconnected with the CeA and insula and has been suggested to act as a relay center that coordinates motor, autonomic and defensive reactions during sustained threat (Davis et al., 2010). Along these lines, the BNST may monitor signals representing threat escalation in the environment (Davis and Whalen, 2001; Walker et al., 2003; Somerville et al., 2010), with a recent functional MRI study by our group showing that the BNST was increasingly activated when subjects monitored the movements (forward vs. backward direction) of a tarantula (Mobbs et al., 2010). Together, the amygdala and BNST may keep track of escalating threat levels (e.g., predator movements). Optogenetic work, however, suggests that the anatomy of the BNST is more complex than previously believed. For example, recent evidence shows that while the oval BNST independently promotes states of anxiety, other anxiolytic roles are found for the anterodorsal BNST (Kim et al., 2013).
- *Safety seeking*. With the added benefit of increasing the likelihood of escape from predators, monitoring the location of safety is one strategy by which to decrease fear. Ecological theorists propose that animals maintain a margin of safety from predators, which is the difference in time to reach cover by prey and predator (Dill, 1990). Elland and Eller have shown that gerbils use the most optimal trajectories to a safe refuge (Elland and Eller, 2009). Safety searching is also particularly prevalent in a number of affective disorders in humans. For example, patients presenting with anxiety disorders, such as panic disorder, generalized anxiety disorder, or agoraphobia may, when provoked, exhibit safety behaviors such as checking the proximity of hospitals and/or building exits (Rachman, 1983, 1984; Kamphuis and Telch, 2000). When under threat, the sight of safety signals reduces fear (Gray, 1971). Indeed, when subjects are placed close to a safety exit, measures of fear decrease (Carter et al., 1995). Safety cues have also been observed to abolish innate defense mechanisms in rodents, such as threat-related analgesia (Wiertelak et al., 1992). One region involved in safety learning may be the vmPFC. Activity in this region is increased in spider phobias after successful reduction of fear via cognitive behavioral therapy (Straube et al., 2006). Others have shown that a safety stimulus during an aversive experience results in increased activity in the vmPFC (Schiller et al., 2010) while decreasing threat also results in increased activity in the same region (Mobbs et al., 2010).
- *Threat value*. How the organism responds to a potentially threatening stimulus is largely dependent on the threat value it ascribes to the stimulus, with the amygdala and vmPFC central to this process. The amygdalae basolateralis (BLA) is suggested to be involved in the encoding of emotional events. For example, researchers have shown that activity in distinct neuronal populations in the anterior BLA coincided with emotional state transitions between high and low fear. Interestingly, these subregions of BLA are connected to the hippocampus and medial PFC (Herry et al., 2008), regions subserving anxiety, memory, and response formation. The CeA, on the other hand, is a major output center, and may be involved in the behavioral expression of fear (Balleine and Killcross, 2006). The CeA may steer threat responses in the midbrain through its connections to the hypothalamus via neuropeptidergic (oxytocin) signaling (Viviani et al., 2011). More recently, exciting research in rodents using optogenetics and electrophysiology has implicated BLA-CeA microcircuits in the control of anxiety (Tye et al., 2011). The amygdala may therefore function as a gatekeeper or switch that first determines the threat value of a stimulus and then coordinates the appropriate reactions to that stimulus, both defensively and viscerally (Price, 2005). While the degree of danger posed by a threat is an important component of models of threat value, another component is implicated in the calculations relating to the likelihood of threat escape by the animal. Here, the PFC may be particularly important, playing a major role in coordinating and instigating responses to risk assessment. Threat exposure tests in rodents have implicated a coupling between the infralimbic (il) PFC and the ventromedial orbital PFC. That is, the ilPFC is involved in the careful evaluation of situations involving threat and the ventromedial orbital PFC facilitates ilPFC-mediated adaptive behavioral responses via inhibition of prepotent avoidant responses (Wall et al., 2004). Input from the ilPFC to GABAergic intercalated cells (ITC) located between the BLA and CeA may inhibit CeA output cells and hence reduce the fear response while input from prelimbic PFC has been shown to have the opposite effect, e.g., facilitation of fear expression (Quirk and Mueller, 2008). As we discuss later, the dorsolateral and ventrolateral zones within the PFC have been associated with the active inhibition of attention toward distractors and emotional reappraisal of prepotent responses to distractors, or cognitive control of emotion (Ochsner and Gross, 2005; Goldin et al., 2008).
- *Predicting the actions of the threat*. Simulation systems support both the prediction (see above) and directed escape strategies of the SOS (Figure 3). Furthermore, action prediction involves producing multiple simulations in order to predict the actions the threat is likely to take, and the ensuing consequences of each action. Therefore, the prediction system plays a critical role in how we respond to threat, especially when the organism has time to choose between various escape options. There may be many possible actions but whether the most optimal action is chosen will depend on the quality of information available to the prey. Cortical brain regions, such as the superior temporal sulcus, have been shown to respond to multisensory information such as sound and vision, (e.g., Hagan et al., 2009), while subcortical regions may be responsible for using multisensory information to cross-validate location, time, sound and identity information (Rao et al., 1997). These regions may support the calculations suggested by Bayesian computational approaches to describe behavior, discussed later.
- *Action preparation and directed escape*. Directed escape is when the organism avoids the post-encounter threat by actively choosing the most appropriate escape actions. Volitional action under predation is likely to involve the neural systems that underlie preparatory motor and aversive systems. Self-initiated actions may be enabled via activation of the supplementary motor area (SMA). For example, cells in the SMA respond to self-initiated movements (Romo and Schultz, 1990), while ablation of the SMA and cingulate motor areas results in disruption to volitional movement (Thaler et al., 1995). Furthermore, the basal-ganglia acts as an interface between emotion and motor responses, thereby playing a key role in action selection and allowing the animal to transform affective information into a motor response (Stocco et al., 2010). Concerning aversive systems, the amygdala is one likely candidate involved in aversive-biased action choice. For example, ablation of the rat CeA suppresses the behavioral responses elicited during exposure to a threatening stimulus, but preserves the ability of the rat to redirect its course of action in order to avoid subsequent exposure to an aversive stimulus. Conversely, ablation of the BLA renders an aversive stimulus unavoidable by the rat, despite the rat exhibiting normal behavioral responses to the feared stimulus (Killcross et al., 1997).

Recent fMRI evidence in humans has shown that the insula is involved in the bodily urge to initiate motor actions (Yu et al., 2010; Mobbs et al., 2013). Furthermore, the vlPFC (which includes right inferior frontal gyrus [IFG]) receives direct input from the striatum (Middleton and Strick, 2000) and is part of the cortico-striatal-pallidothalamocortical loop. Research in non-human primates also suggests that the lateral PFC is involved in planning future actions (Mushiake et al., 2006). In humans, a diffusion tensor imaging (DTI)/fMRI study by Aron and coworkers showed the right IFG to be part of a wider cognitive control network that included the subthalamic nucleus and pre-SMA (Aron et al., 2007). Other connections of the IFG, such as those to the ACC and pre-SMA, may play a role in shifting attention and in the initiation and suppression of movement (Garavan et al., 1999). Dorsal parts of the ACC (dACC) are connected to the premotor cortex and amygdala (Bates and Goldman-Rakic, 1993) and, as such, are thought to modulate both the control and monitoring of action (Amodio and Frith, 2006).

### Defensive strategies

Defensive strategies instantiated in the midbrain and hypothalamus are typically evoked during circa-strike attack. A fast-acting reaction system has obvious evolutionary benefits and perhaps, not surprisingly, this reaction system resides in the oldest regions of the human central nervous system. Price points out that the first mammals were often preyed on by reptiles and birds and consequently the mammalian brain evolved to enable quick instinctive reactions (Price, 2005). Immediate threat responses were, and continue to be, hard-wired spinal reflexes that provide rapid reactions to threat (Lee et al., 1996). The FFF-system also provides fast reflexive responses, but tends to be more sophisticated. For example, when under direct attack (i.e., circa-strike), the animal will try to escape the situation. Escape in this context is therefore indirect where the organism attempts to terminate exposure to the attacking (or perceived attacking) threat, yet has limited plan of action (Gray, 1971). If escape is unsuccessful, the animal will either resort to fighting or “playing dead.”

The PAG is the core neural substrate involved in FFF-behaviors. The PAG is a midbrain structure that encircles the cerebral aqueduct (Bandler et al., 2000) and can be divided into four structurally and functionally distinct neuronal columns; the dorsomedial, dorsolateral, lateral and ventrolateral aqueduct (Bandler et al., 2000). Rodent studies show that mere exposure to a predator leads to immediate increases in cFos gene expression in the rostral dorsomedial/dorsolateral PAG (dm/dlPAG; Bandler et al., 2000). Human fMRI studies also show a switch from the ventral prefrontal cortex to the midbrain PAG area the closer a threat moves towards its target (Mobbs et al., 2007, 2009, 2010), thereby supporting the role of the PAG in negotiating highly imminent threat. More specifically, the PAG is responsible for at least two evolutionary conserved types of coping behaviors:

- *Passive coping*. Passive coping is typically associated with freezing during threat detection and assessment and is instigated by the vlPAG and its connection with the medial CeA (Bandler et al., 2000). A recent optogenetic study showed that inhibitory microcircuits in the lateral subdivision of the CeA gate the medial CeA output, thereby controlling conditioned freezing responses (Haubensak et al., 2010). Recent models of freezing hold that different subtypes of freezing behavior are evoked by distinct columns within the PAG, and that each freezing subtype serves a different function (Brandao et al., 2008). For example, Brandao et al. have shown that stimulation of the dPAG elicits several types of freezing that differ in terms of freezing onset time: (i) dPAG-evoked freezing where freezing occurs during low intensity electrical stimulation; and (ii) dPAG post-stimulation freezing, where freezing occurs after electrical stimulation. Furthermore, stimulation of the vPAG results in a form of freezing that is associated with the recovery element of the defense–recuperative process. Freezing induced by either dPAG or vPAG stimulation is hypothesized to occur when threat is highly imminent or when the animal has been attacked. Thus, freezing may take several forms, some enacted by post-encounter threats, others when the threat is high in intensity such as that experienced during circa-strike threat.
- *Active coping*. Fight and flight are examples of active coping strategies and are mediated by the dmPAG and dlPAG. For example, microinjections of excitatory amino acids placed in caudal portions of the dlPAG result in flight behaviors (Keay and Bandler, 2002) and chemical stimulation of the dlPAG elicits uncoordinated panic-like behaviors such as vigorous running and jumping (Deakin and Graeff, 1991; Bandler et al., 2000; Vianna et al., 2001). Moreover, lesions to the dlPAG eradicate such bursts of activity (Depaulis et al., 1989). In humans, activity in the PAG is observed during highly proximal threat (Mobbs et al., 2007, 2009, 2010) and electrical stimulation of the PAG results in panic-like symptoms, including feelings of terror, a desire to escape the situation, and hyperventilation (Nashold et al., 1969). In humans, panic symptoms are thought to be mediated by the dPAG and panic disorder patients show abnormal gray matter density in this region (Uchida et al., 2008). Panic disorder is proposed to be associated with the dorsal raphe nucleus (DRN)-periventricular pathway which includes projections between the hypothalamus and PAG (Deakin and Graeff, 1991). Others suggest that the PAG detects respiration and panic results from feelings of suffocation (Schimitel et al., 2012).

The hypothalamus and PAG are key brain regions involved in the fight responses that occur as a last ditch attempt to deter predators. Stimulation of the hypothalamus in cats elicits “sham rage” responses, including back arching, ear lowering, piloerection, striking and hissing behaviors (Reis and Fuxe, 1969). Lin et al. used optogenetic techniques to show that stimulation of the ventrolateral subdivision of the ventromedial hypothalamus in rats results in the attack of conspecifics and inanimate objects (Lin et al., 2010). The dPAG is directly connected to the hypothalamus and is critical for active coping behaviors including fight responses (Bandler et al., 2000). Stimulation of the PAG has been shown to elicit aggressive behavior (Potegal et al., 1996). It has been suggested that the PAG, amygdala and hypothalamus together may comprise a rage circuit (Panksepp, 1998). Indeed, the PAG has been suggested to play a role in evaluating the emotional content of frustrating events, perhaps, through its connections with hypothalamus, medial PFC, and insular cortex (Bandler, 1988).

#### Escapability

The presence of an escape route may, in part, determine an animal's decision to either freeze or flee from danger (Blanchard and Blanchard, 1989). Studies of learned helplessness in rodents demonstrate that, when faced with inescapable electric shocks in one environment, rodents do not attempt to escape electric shocks in a different environment (Seligman and Maier, 1967). Neurobiological, combined with pharmacological, work has shown that the DRN receives signals about the controllability of the threat from infralimbic and prelimbic sectors of the ventral mPFC in rodents and that Muscimol knockout of this region results in indiscriminate firing by the DRN (Amat et al., 2005). Interestingly, in healthy humans, the mPFC, including the rostral ACC, becomes active when subjects are chased by a highly inescapable threat (Mobbs et al., 2009). The DRN, which contains around 30,000 neurons in the rodent, is unlikely to modulate complex processes (Maier and Watkins, 2005), yet it receives dense connections from regions where complex signals are processed in the forebrain, such as the amygdala and PFC (Amat et al., 2006). Moreover, exogenous opioids activate DRN 5-HT neurons by inhibiting gamma-aminobutyric acid (GABA) receptors at neurons within the DRN. The DRN is a major component of the 5-HT system and, as a site of action for serotonin-specific reuptake inhibitors (SSRIs), is thought to be critical in the pathogenesis of anxiety and panic (Deakin and Graeff, 1991; Maier and Watkins, 2005).

#### Preparing for contact: analgesia

The importance of analgesia is clear—it facilitates escape by reducing pain in the injured animal. The alternative is that the animal is unable to move due to intense pain or tissue damage, and will most likely die as a consequence. Analgesic systems are likely to be evoked prior to attack, for example, when the animal is pursued by a predator. The PAG, together with the dorsal horn of the spinal cord and several cortical areas (Petrovic et al., 2002), also plays a key role in eliciting endogenous opioid and non-opioid analgesia. Analgesia might require imagined or anticipatory activation of the US representation in order to prepare the organism for a nociceptive outcome (Hollis, 1982). This process, which accompanies the Post-Encounter stage, is thought to evoke the endogenous opioid system (Fanselow and Lester, 1988). Stimulation of the rodent lateral or dorsal PAG results in heightened threat and non-opioid-dependent analgesia (Comoli et al., 2003). In humans, the PAG is active during both placebo and opioid analgesia (Petrovic et al., 2002), suggesting that the basis for placebo effects might lie within the brain's ability to anticipate injury.

For the Defense System, the “behavioral options” are clearly within the hard-wired FFF-complex and involve escape reflexes. The “cognitive operation” is limited in the sense that there is no time for the cognitive system to have a significant influence (e.g., “strategic” decision making during indirect escape). However, it is plausible that faster “intuitive” cognitive systems may optimize the organism's response to imminent threat. The “strategic” cognitive system could also help by providing learned information about the environment, including escapability, and allow for the control of behavior or affect. The degree of affect and the ability to regulate it may determine the amount of cognitive flexibility available (being cool under pressure vs. fighting or fleeing). One interesting proposal is that individual differences in these different proclivities will relate to psychopathology.

## Modulatory and learning systems

We propose that survival strategies are influenced by two core systems. (i) Modulatory systems include cognitive appraisal/regulation, memory of encounter situations, interoception, motivation, metabolic needs, and the functioning of domain-general cognitive machinery which is integrated at both the emotional regulation and strategy; and (ii) Learning systems include internal probabilistic models and vicarious learning. Modulatory systems have direct control over the five survival strategies of the SOS. On the other hand, the learning system optimizes survival strategies and modulatory systems by providing probabilistic information and drawing on other information that is relevant to the situation (i.e., learning about a threat from others). Modulatory Systems can override Learning Systems. For example, our own personal encounters can override those learned vicariously.

### Modulatory systems

Given the sheer diversity of threat humans encountered on their voyage to conquer the world, it is clear that we needed a flexible system that controls how we appraise (e.g., Mobbs et al., 2006; Barrett and Kensinger, 2010) and manage threat responses. An efficient system should flexibly tailor responses to specific circumstances via a set of ecologically-learned actions which would confer the greatest benefit to the survival of species. According to the “risk allocation hypothesis,” risk of predatory attack changes over time and this has a direct effect upon intensity of vigilance and time spent foraging (Lima, 1990).

### Reappraisal and suppression

Two ways of actively controlling a survival behavior are through suppression (actively keeping threat or information about threat out of mind) and reappraisal—actively changing the way one thinks about a threat or information pertaining to threat. While most prominent during prediction and threat assessment, these deliberate conscious processes clearly influence all survival strategies of the SOS, albeit with decreasing efficacy as one moves down the defense gradient. Indeed, Ohman and Mineka point out that automatic responses, such as those instantiated in the FFF-system, are likely to be impenetrable to conscious cognitive control. In the face of this decreasing control, there is little doubt that conscious reappraisal would play a major role in both prediction and threat assessment systems when the threat is distal and there is time to think. Candidate neural regions involved in cognitive appraisal include dorsomedial, ventrolateral (vl) and dorsolateral (dl) PFC. The vlPFC has been suggested to be involved in reappraisal of emotion (Wager et al., 2008) and control of attention (Ochsner and Gross, 2005). The dlPFC has been implicated in cognitive operations such as behavioral selection, top-down control of memory, attention maintenance and suppression of unwanted memories (Anderson and Phelps, 2001; Baird and Fugelsang, 2004; Gordon et al., 2004; Bechara and Van Der Linden, 2005). In relation to the rostro-caudal model of cognitive control, the dlPFC has been implicated in sensory and contextual levels of control (Badre and D'Esposito, 2009). Given these roles, the dlPFC is an important part of the behavioral inhibition circuit that controls prepotent emotional responses, otherwise known as a cortico-striatal-thalamocortical loop (Masterman and Cummings, 1997; Owen et al., 1998; Middleton and Strick, 2000). Systems that detect and resolve conflict represent an important extension of top-down control processes by PFC (Cavanagh et al., 2013). Again, these processes would presumably be highly beneficial during threat assessment.

### Regulation

Closely allied with the active processes of reappraisal and suppression is the ability to passively regulate the survival circuit. A candidate region for this operation is the subgenual ACC (sgACC)—a region known to play a role in mood regulation, extinction, stress responses and learned fear (Phelps et al., 2004; Mayberg et al., 2005; Corcoran and Quirk, 2007). Interestingly, the sgACC is connected to the amygdala and other parts of the threat circuitry (i.e., PAG) and sgACC activity has been shown to be disrupted in many affective disorders such as depression, bipolar disorder and PTSD (Price and Drevets, 2010). Mayberg et al. (2005) conducted a cutting-edge study whereby electrodes were implanted in the sgACC of patients with treatment-resistant depression. Following stimulation of the sgACC, remission of depression was observed in the majority of patients (Mayberg et al., 2005). Mayberg et al. work has since been supported in rodents using optogenetic methods, thus further supporting the role of the vmPFC (including the sgACC) in the control of emotion (Covington et al., 2010). More recently, we have shown that when subjects are trained using an emotional working memory task, they become better at reappraisal when viewing negative stimuli. This increased ability is predicted by increased activity in the fronto-parietal network and the sgACC (Schweizer et al., 2011, 2013). Although more research is needed, this suggests that the sgACC interacts with cognitive networks involved in affective regulation and suppression.

### Interoception and feeling

Signals from physiological and hormonal systems serve as critical contexts (Maren et al., 2013) influencing optimal decision-making (Damasio, 1994). Critchley and Nagai (2012) suggest that: “The changing representation of internal bodily ‘self’ exerts a dynamic contextual effect on emotional processing.” Furthermore, there is consensus on how people rate their bodily sensations of fear and anxiety (Nummenmaa et al., 2014), yet these sensations can be misinterpreted. For example, Schachter and Singer demonstrated that when people are given a sham vitamin injection (which in reality was a norepinephrine agonist that increases autonomic arousal), the cognitive labeling of their emotion experience was altered. As Schachter and Singer explained, subjects search the context for an explanation of their physiological experiences, irrespective of whether the context was responsible for generating those experiences (Schachter and Singer, 1962). The dACC activates during sympathetic-excitation including heart rate, blood pressure and pupil dilation (Critchley et al., 2001), while the insula may generate an autonomic signal used to promote avoidance of possible aversive outcomes (Yu et al., 2010).

Research has shown individual differences in the ability to detect one's internal bodily state. When Critchley et al. asked subjects to detect their heart rate, they found that when they compared interoceptive (detecting one's heartbeat), to exteroceptive attention (detecting different notes), there was increased neural activity in the bilateral region of the anterior insula. The authors suggest that the anterior insula is involved in the subjective awareness of visceral feelings (Critchley et al., 2004). The importance of interoception on decision-making has been exemplified by Dunn et al. (2010) who extended on these early studies by showing that participants who were better at keeping track of their heartbeat, were better at tracking their subjective arousal ratings and this ability facilitated intuitive decision-making.

### Other survival considerations: metabolism, energy, reward, and homeostasis

Critical to the SOS is the idea that animals have to weigh the risk of predation with a need to satisfy their metabolic needs (Sapolsky, 2004; Barrett, 2005). These two pressures are in direct competition with each other. Closely related to this phenomenon is Lima et al. (1985) energy-predation risk trade-off model. Their model was based on the observation that ground squirrels sometimes eat food immediately when the threat of predation is high, but other times take food to the safety of their tree. The latter strategy is inefficient in the sense that more energy is consumed when carrying food back to safety—energy that could be used to escape predators. While the authors supported this trade-off, they also found that when the food was close to the tree, the squirrels would carry the food back to the tree, suggesting that when energy consumption was relatively low, the optimal behavior was to prioritize safety. However, studies show that hunger changes the dynamics of the energy-predation risk trade-off (Sih et al., 1988). Milinski and Heller (1978) showed that hungry Sticklebacks will change feeding behavior in an effort to maintain predator vigilance. This cost-benefit behavior extends to arthropods. For example, hungry spiders (*Pardosa Milvina*) show a pattern of consuming the same amount of food when under predation, compared to non-predation. However, when sated, they consumed significantly fewer prey when under predation risk (Persons et al., 2002). Although little work has been carried out in humans, Symmonds et al. (2010) showed that subjects' risk preferences were mediated by their metabolic state. Furthermore, people make poorer health judgments when hungry (Tal and Wansink, 2013). Together, it seems that when metabolic needs are high and when starvation is imminent, the SOS would be reconfigured to aid the immediate optimal survival response (e.g., consumption of food).

## Learning systems

While innate neural mechanisms prepare us to react to ecological dangers, our experiences are responsible for shaping how we navigate the world. A critical component of the SOS is the ability to continually update and modify responses through learning (e.g., outcome evaluation and prediction error). Flexible learning systems modulate both soft-wired higher order systems such as prediction, and basic associative systems (i.e., Pavlovian and instrumental conditioning) including hard-wired attention, autonomic and flight, fight and freezing (FFF)-responses. While associative learning is a component at all levels of the SOS, more complex predictions and simulations involve probabilistic models that increase the effectiveness of behavioral responses. Probabilistic models can be learned and refined via direct experience with threats or by observing others under attack.

### Encounter (face-to-face) learning

Threat encounters that do not result in death provide valuable information that results in an increased ability to avoid future threats with specific predators. Associative learning is conserved across most species, providing quick and decisive actions based upon experience. Given its importance and effectiveness across evolutionary history, this type of learning plays a role in all levels of the SOS model, influencing and guiding many behavioral actions. For instance, animals will associate a particular context, event or particular actions with threat. These features become bound together such that future encounters with a similar context, event, or particular actions evoke representations of their consequences, resulting in physiological changes and prompting the organism to action. This type of learning is highly adaptive, quickly generating a more accurate representation of the world and its potential threats in order to optimize survival behaviors.

Simple computational models proposed by associative learning theories are effective in describing how organisms learn about threats. Pavlovian conditioning involves the strengthening of a stimulus-outcome association, while instrumental conditioning involves the strengthening or weakening of a behavioral action based on its consequences. These simple types of learning can be further enriched, For example, in Pavlovian-Instrumental Transfer (PIT) an instrumental behavior is influenced by a Pavlovian stimulus. For example, PIT occurs when the animal hears the sound produced by the predator, and then further avoids the predator's territory. PIT effects can be specific or general, depending on whether the Pavlovian stimulus influences the vigor of responding of an instrumental action previously paired with the same or different outcome as the stimulus. In both rodents and humans, these two forms of PIT have been shown to rely on different sub-regions of the amygdala (Corbit and Balleine, 2005; Prévost et al., 2012) and the striatum (Bray et al., 2008; Talmi et al., 2008; Corbit and Balleine, 2011).

Computational models of increasing complexity have been developed within the theoretical framework of associative learning. These models make various assumptions about the strength of associations between stimuli and outcomes based upon the sum total of coincidences leading up to a current state. Subsequent pairings serve to update the organism's expectation or likelihood that a particular outcome will occur given a particular event or behavior. The most influential of these models was proposed by Rescorla and Wagner (Rescorla and Wagner, 1972), which argues that the strength of the conditioned response (e.g., freezing) depends on the associative strength of the CS (e.g., the sound produced by the predator). In this model, the change in associative strength ΔV for stimulus A (ΔVA) on a given trial, is described by the following equation:

ΔVA=αβ(λ−VT)

The change in associative strength is directly related to the difference between the magnitude of the actual outcome λ, and the associative strengths of all other CS's present on that trial (VT; also called prediction error), and modulated by two learning-rate parameters that take values between 0 and 1. The value of α is determined by the salience of the CS, and that of β by characteristics of the unconditioned stimulus (US). This model generates two computational signals: expected value signals (i.e., “what do I expect from this particular stimulus or action?”) and prediction errors (PE) that update expectations (i.e., “what did I receive compared to what I was expecting to receive?”). Organisms can use these signals to indicate the magnitude of threat and guide subsequent behavior. For example, if a particular sound produces a strong expected threat signal, but the threat does not materialize, a PE signal will be generated. The PE signal will influence future behavior when the same sound is encountered, modulated by the current strength of the association. Thus, online updating of learned associative information through these signals is a highly adaptive process that enhances survival behaviors, including actions that result in swift reduction of threat. Several studies suggest these signals and their subcomponents are represented in a number of brain regions in humans and animals, most notably the amygdala, the ventral striatum, the medial PFC, OFC, and VTA (Fanselow and LeDoux, 1999; Buchel and Dolan, 2000; Elliott et al., 2004; O'Doherty et al., 2004, 2007; Seymour et al., 2005; Yacubian et al., 2006; Hampton et al., 2007; Prévost et al., 2011). Until recently, appetite and aversive PE signals were assumed to be integrated and underwritten by a single system in the brain. However, recent findings suggest that aversive PEs occur in distinct areas outside the brain's dopamine driven reward system. A pair of studies found evidence for aversive PE signals in the amygdala and PAG (Roy et al., 2014), challenging the commonly held assumption that a single system processes both reward-based and aversive PEs. These findings highlight the flexibility of neural circuits and the functions they can subserve, and suggest that more complex and sophisticated sets of computations can be carried out by phylogenetically older neural structures. This flexibility may also be responsible, in part, for behavioral adaptability observed in natural ecology.

Another model predicts that attention to a stimulus will decrease over time to the extent it proves to be a good predictor of reinforcement (Pearce and Hall, 1980). Indeed, the Pearce-Hall model assumes attention to a stimulus is necessary during early associative learning and encoding, but not required once a stable association has been established. In this model, VT represents the associative strength of all CSs present on the trial, as in the Rescorla-Wagner model. However, rather than calculating a change in associative strength, the Pierce-Hall model solves for the associability, α, of a stimulus A on a trial n, or αAn, by subtracting VT from the intensity of the US. Neuronal activity in both the rodent and human amygdala is associated with the associability signals calculated using this model (Roesch et al., 2010).

$$\alpha \text{An}=\;|\lambda -\text{VT}{|}_{\text{n}-1}$$

High values of the quantity λ − VT correspond to a high quantity of information. A greater amount of information is conveyed when observing unexpected outcomes than expected outcomes, especially in circumstances in which uncertainty is high. The PE value has an analog in information theory, in which information quantity can be characterized as a measure of entropy, *H*(*x*) = −Σ*p*(*x*) log *P*(*x*). Every source has associated with it a degree and kind of uncertainty that is either expected (known range of unreliability) or unexpected (unknown, or violations of expectations), and quantified by a value of entropy. Entropy from a source constrains the amount of information that can be communicated by it, and is related to the uncertainty inherent in the timing of the US. The CS conveys information about the US by reducing uncertainty. As a general rule outcomes that are unexpected, i.e., have a low subjective probability of occurring according to an internal model of an organism, reduce uncertainty, convey more information than expected outcomes (when λ —VT is small and *H*(*x*) is large) and may have a greater effect on subsequent behavior or responses. In ecological terms, large values of the modulus of the differential correspond to potentially important information that should result in immediate direction or redirection of attentional resources to the stimulus in order for the organism to evaluate the magnitude of threat. This process drives the strength of associability encoding of stimulus and outcome, which is used by the organism to guide behavior when it encounters the same stimulus. In this model, changes in the modulus will interrupt ongoing behavior in order to increase vigilance, providing a threat reduction mechanism and enhance survival behaviors. However, while this model captures learning about associations, it fails to explain attentional redirection and vigilance behaviors for high threat and highly reliable associations that do not decrease in strength.

These models provide formal explanations for learning that takes place during Pavlovian and instrumental conditioning and are supported in both animal and human literature (Pearce and Bouton, 2001; Delgado et al., 2008). Human fMRI has revealed the nucleus accumbens (NAcc) to be activated during aversive conditioning when pharmacologically manipulated with a dopamine agonist (Menon et al., 2007). Interestingly, the BLA has been implicated in attention (Roesch et al., 2012) while the OFC and NAcc may relate to information about expected values and outcome expectation (Roesch et al., 2010; Li et al., 2011). However, these and other derivative models fail to predict outcomes in changing contexts and circumstances, notably exploratory behavior, novelty, uncertainty, and extinction (Miller et al., 1995; Phelps and LeDoux, 2005; Dayan and Niv, 2008).

More recently, a “Hybrid model” that combines features of the Rescorla-Wagner and Pearce-Hall models was developed in the fear conditioning literature in humans (Li et al., 2011). As expected, the amygdala was involved in tracking associability in their task and the ventral striatum implemented reinforcement prediction errors. Another class of models using Bayesian inference known as “model-based” models, involve more sophisticated representations and computations about the world. This class of models assumes that the nervous system maintains internal probabilistic models used to generate predictions about the world, which are updated as the organism encounters and accumulates external sensory information (see Knill and Pouget, 2004). Such a mechanism would be particularly valuable to niche-independent animals where survival depends on fast and accurate responses to newly encountered dangers. Bayesian models, in which the organism uses available evidence to build a model and fine-tune its knowledge of the world and how it works, differ markedly from association-based models, in which the animal learns through crude associations. Incoming multi-modal sensory information is comprised of noise and signals of interest, and must be processed effectively by the brain to produce appropriate behaviors. Bayesian models examine the reliability of information coming from a particular source and to a particular sensory modality, and are used to integrate this information via a weighted sum of the most likely outcomes (i.e., the direction of a threat). This class of model can handle contexts in which an organism is presented conflicting information and must use the available information to make optimal behavioral decisions. The ability to effectively handle ambiguous, or uncertain sensory information is critical in natural ecology to avoid and limit threat encounters. Emerging evidence suggests that Bayesian models capture learning in both instrumental and Pavlovian settings as well, and in some instances perform better than association-based models (Prévost et al., 2013). The Bayesian framework has been applied to model cognitive processes and learning ranging from perception and attention to sensorimotor learning (Yu and Dayan, 2005). Richer internal models and representations have an advantage over less sophisticated associative models in that they enable a greater degree of flexibility in behavior due to their ability to make generalized predictions, i.e., about classes of stimuli.

All of the models discussed above may be realized in different contexts and utilized for specific survival purposes in natural ecology. Both the Rescorla-Wagner and Pearce-Hall models assume a basic mechanism through which to avoid threat, while Bayesian models assume a much more complex representation of the possible dangers present in one's environment. In the former models, feedback from threat situations is used to update knowledge, with a particular emphasis on the associability or relevance of the threat stimulus that will further strengthen associations and therefore learning about potential dangers in the case of the Pearce-Hall model. On the other hand, potential threats and the probability of their occurrence will be anticipated and predicted when Bayesian inferences are used and might therefore be more optimal for threat avoidance and ultimately survival. These models would therefore be evoked during the different survival contexts laid out by the Threat Imminence, and Lima and Dill models. For example, Bayesian models would be utilized during the prediction and pre-encounter phases, Rescorla-Wagner and Pearce-Hall models during post-encounter threat orienting and circa-strike.

### Vicarious learning

Learning about threats via direct encounters is effective but involves increased risk of death. A less dangerous and therefore more optimal learning method is witnessing the experiences of others. According to “Social Learning Theory,” knowledge transfer depends on the willingness of the teacher to teach the desired skill, and the ability of the learner to receive and retain information, and to reproduce the behavior (Bandura, 1962). In a classic study, Mineka et al. showed that socially transmitted fear is critical to non-human primate learning (Mineka et al., 1984). Likewise, humans and other animals will seek advice from others when navigating unknown and unpredictable environments. Vicarious learning has been observed in a variety of animals from drosophila to mice. For example, a recent study showed that “observer” mice learn vicariously through other “demonstrator” mice when to freeze, and inactivation of the ACC impaired these vicarious responses (Jeon et al., 2010). Another study featured birds trained in captivity to perform an action required to solve a puzzle and find food. These birds were then released into the wild for observation. Other birds not trained quickly learned the adaptive behavior and were able to perform the same action to find the food (Aplin et al., 2014). This suggests that vicarious learning is more prevalent than once believed.

In humans, vicarious learning is certainly more sophisticated and transmitted through multiple channels. These channels include conversation, reading or other modern-day media such as radio, television, and the internet. Human behavioral and brain imaging studies have shown that the vicarious transmission of fear is mediated by the amygdala, ACC, and medial PFC (Olsson et al., 2007; Olsson and Phelps, 2007), yet little is known about the mechanisms. Price and Boutilier (2003) have put forward a “Bayesian Imitation Model,” stating that we combine the information learned through the observation of others with the existing knowledge and data obtained through our own personal experiences. While research into the computational mechanisms associated with vicarious learning is ongoing, observational learning may be further explained by what Burke and coworkers call “observational action prediction errors” and “observational outcome prediction errors,” represented in dlPFC and vmPFC, respectively (Burke et al., 2010). It may be that these neural systems not only allow us to vicariously learn about threats, but also to update our internal probabilistic models and the PFC may play a critical role in such operations. Intriguingly, the neural systems associated with vicarious learning are connected to other systems that underlie social behavior and imagination, including the STS, temporal parietal junction (TPJ), and PCC. Although one can only speculate, it is interesting that the STS, TPJ and PCC are connected to the mPFC network proposed by Price (2005) suggesting that complex neural systems are connected to older defensive systems.

### Bridging data: inference and symbolic thought

Humans possess an impressive ability to collate and bridge information across numerous experiences and form new higher-order representations. Inference allows one to produce new beliefs, expectations and plans based on combining existing knowledge (Mercier and Sperber, 2011). Penn et al. (2008) suggest that such inferences can be based on hierarchical relations and extend to unobservable cases such as others' mental states or the strategies of the potential threat. With these tools, humans can simulate the actions of a threat and integrate this with other information streams to rationally build new information about the threat and anticipate ways to reduce future deadly encounters. For example, theorists point out that only humans can reinterpret the world via hypothetical and unobservable entities (Povinelli et al., 2000) and this ability builds a capacity for symbolic relationship between simulating (unobservable) future threat outcomes and ways to prevent such dangers by changing behavior. Although the exact mechanisms are still disputed (c.f. Penn et al., 2008), theorists maintain that symbolic thought is part of a domain-general operator that links many cognitive modules and organizes them into a single “object file” (Kahneman et al., 1992).

## Concluding remarks

Evolution is the one true incremental scientist and the human brain is one of its greatest accomplishments. Using theoretical models from behavior ecology and behavioral neuroscience, we propose five strategies that are central to survival: prediction and prevention occur during pre-encounter threat; orienting toward threat and threat assessment occur during post-encounter and rapid reaction to imminent danger occurs during circa-strike attack. Furthermore, these strategies are influenced by many intervening variables including a modulatory system that directly influences survival strategies by actively reconfiguring the threat circuits. Feeding into these strategies and modulatory systems are a set of learning systems, which range from simple associative learning to higher-order inferences. Supporting the SOS is a circuit extending from interconnected cortical-hippocampal systems that support the prediction and simulation of situations through to the PFC, amygdala and BNST associated with threat assessment and defensive FFF-behaviors in the PAG and hypothalamus. While humans are confined to many of the same evolutionary stable strategies that govern other living organisms (Gray, 1971), we propose that the human SOS is enhanced in the following operations:

- An imagination system characterized by rich representations and capable of sophisticated simulations, including future threat encounters.
- The ability to reduce threat by changing the environment (niche construction) and forming social coalitions.
- The flexibility to willfully inhibit-excite survival strategies.
- To learn vicariously about threats and empathize.
- The ability to symbolically reason and bridge data to build new information about threats.

Other animals possess rudimentary features of these operations, yet the enhanced features of the human SOS allow us to ruminate about the past and envision worrying futures. In the absence of threat, both prediction and prevention strategies allow us to minimize future interactions with danger. For example, in face-to-face encounters, the tiger always wins and a system that minimizes direct contact with life-endangering predators will always be advantageous. It is also plausible that selection pressures favored large cooperative social networks and niche construction, both protecting us from a diverse array of threats and allowing us to evolve perform other survival activities important behaviors (e.g., farming). Conversely, these enhanced abilities may leave us open to affective disorders that involve anticipation or uncertainties concerning the future (e.g., anxiety), sensitivity to social rejection and the fear of leaving the safety of our protective environment.

### Conflict of interest statement

The authors declare that the research was conducted in the absence of any commercial or financial relationships that could be construed as a potential conflict of interest.
